# Conserved IQ motifs, divergent apo‐calmodulin recognition in UBE3B and UBE3C

**DOI:** 10.1002/pro.70826

**Published:** 2026-10-23

**Authors:** Emily G. Schaffter, Xiaohuan Lu, Anna E. Tinklenberg, Megan G. Hill, Rafael H. Levin, Javier F. Tabima, Shuanghong Huo, Donald E. Spratt

**Affiliations:** ^1^ Gustaf H. Carlson School of Chemistry and Biochemistry Clark University Worcester Massachusetts USA; ^2^ Department of Biology Clark University Worcester Massachusetts USA

**Keywords:** apo‐calmodulin, calmodulin, HECT E3 ubiquitin ligase, IQ motif, isothermal titration calorimetry, molecular recognition, NMR spectroscopy, protein evolution, UBE3B, UBE3C

## Abstract

Calmodulin (CaM) regulates diverse signaling proteins through recognition of short linear motifs, including isoleucine‐glutamine (IQ) motifs that commonly bind Ca^2+^‐free CaM (apo‐CaM). The homologous to E6AP carboxyl terminus (HECT) E3 ubiquitin ligases ubiquitin protein ligase E3B (UBE3B) and ubiquitin protein ligase E3C (UBE3C) are closely related enzymes that both contain *N*‐terminal IQ motifs, yet whether these motifs encode conserved modes of apo‐CaM recognition has remained unclear. Here, we combined isothermal titration calorimetry, nuclear magnetic resonance (NMR) spectroscopy, and computational modeling to compare apo‐CaM binding by the UBE3B and UBE3C IQ motifs. The UBE3B IQ motif bound apo‐CaM with high affinity and preferentially engaged the CaM *C*‐lobe, whereas the UBE3C IQ motif interacted more weakly under the same conditions. NMR titrations revealed distinct molecular recognition modes—UBE3B IQ binding occurred in slow exchange and produced a localized *C*‐lobe interaction surface, while UBE3C IQ binding occurred in fast exchange and generated smaller, more distributed chemical shift perturbations. Structural models and molecular dynamics simulations provide a framework for interpreting these divergent apo‐CaM engagement modes despite conservation of canonical IQ motif features. Phylogenetic analysis further suggests that the UBE3B and UBE3C IQ motifs have followed divergent evolutionary trajectories within the broader IQ motif family. Together, these findings show that conserved IQ motifs in related HECT ligases can encode different apo‐CaM binding modes, providing a molecular basis for divergent CaM‐mediated regulation of UBE3B and UBE3C.

## 1 INTRODUCTION

Ubiquitin protein ligase E3B (UBE3B) and ubiquitin protein ligase E3C (UBE3C) are closely related homologous to E6AP carboxyl terminus (HECT) E3 ubiquitin ligases that catalyze ubiquitin transfer through a conserved *C*‐terminal HECT domain (Fokkens et al., 2026; Huibregtse et al., 1995; Lorenz, 2018; Rotin & Kumar, 2009; Wang et al., 2020). This catalytic domain contains an *N*‐lobe that recruits a ubiquitin‐charged E2 conjugating enzyme and a *C*‐lobe that contains the catalytic cysteine required for ubiquitin transfer (Kamadurai et al., 2009). In contrast, the *N*‐terminal regions of HECT ligases are more variable and often contribute to substrate recognition, localization, and regulatory control.

Recent cryo‐EM studies of full‐length and near full‐length HECT ligases have reinforced the idea that HECT activity is regulated through family member‐specific domain architectures rather than a single conserved autoinhibitory mechanism. For example, recent UBR5 structures revealed that its HECT domain is embedded within larger oligomeric assemblies rather than functioning as an isolated catalytic module. These scaffold‐like quaternary assemblies position the HECT domain within a larger structural framework and appear to regulate ubiquitin chain elongation through long‐range intramolecular organization (Hehl et al., 2024; Hodakova et al., 2023; Wang et al., 2023). Recent structures of HECTD3 in apo and ubiquitin‐loaded states further showed that ubiquitin transfer is coupled to conformational rearrangements within the catalytic region (Huber et al., 2026). Structural studies of HUWE1 similarly revealed that its large *N*‐terminal solenoid architecture organizes the HECT domain and is required for full ligase activity (Grabarczyk et al., 2021; Hunkeler et al., 2021). Taken together, these studies highlight a central phenomenon of the HECT family function: although the catalytic HECT domain is conserved throughout the family, unique regulatory mechanisms are often encoded by divergent regions *N*‐terminal to the HECT catalytic core.

Despite these family‐wide differences in regulation, UBE3B and UBE3C share a similar overall domain organization, with an *N*‐terminal isoleucine‐glutamine (IQ) motif and a *C*‐terminal HECT domain (Figure 1), yet appear to function in distinct biological contexts. UBE3B has been associated with neuronal development, mitochondrial function, and cellular responses to oxidative stress (Braganza et al., 2017), whereas UBE3C has been linked to proteasome function, protein quality control, autophagy, interferon signaling, and cancer‐related pathways (Cui et al., 2024; Sun et al., 2024; You & Pickart, 2001). Their disease associations also differ, with pathogenic variants in *UBE3B* genetically linked to Kaufman oculocerebrofacial syndrome (Basel‐Vanagaite et al., 2012; 2014; Flex et al., 2013; Yilmaz et al., 2018), while disruption of UBE3B function has also been associated with neurodevelopmental abnormalities including intellectual disability and autism‐related phenotypes (Cheon et al., 2019). In contrast, UBE3C has primarily been linked to proteostasis pathways, cancer biology, and protein quality control mechanisms (Sun et al., 2024; You & Pickart, 2001), while emerging genetic evidence suggests a possible role for *UBE3C* in neurodevelopmental disorders with autism‐related features (Faqeih et al., 2023). These differences suggest that UBE3B and UBE3C may have evolved distinct regulatory mechanisms despite their shared domain architecture.

**FIGURE 1:**
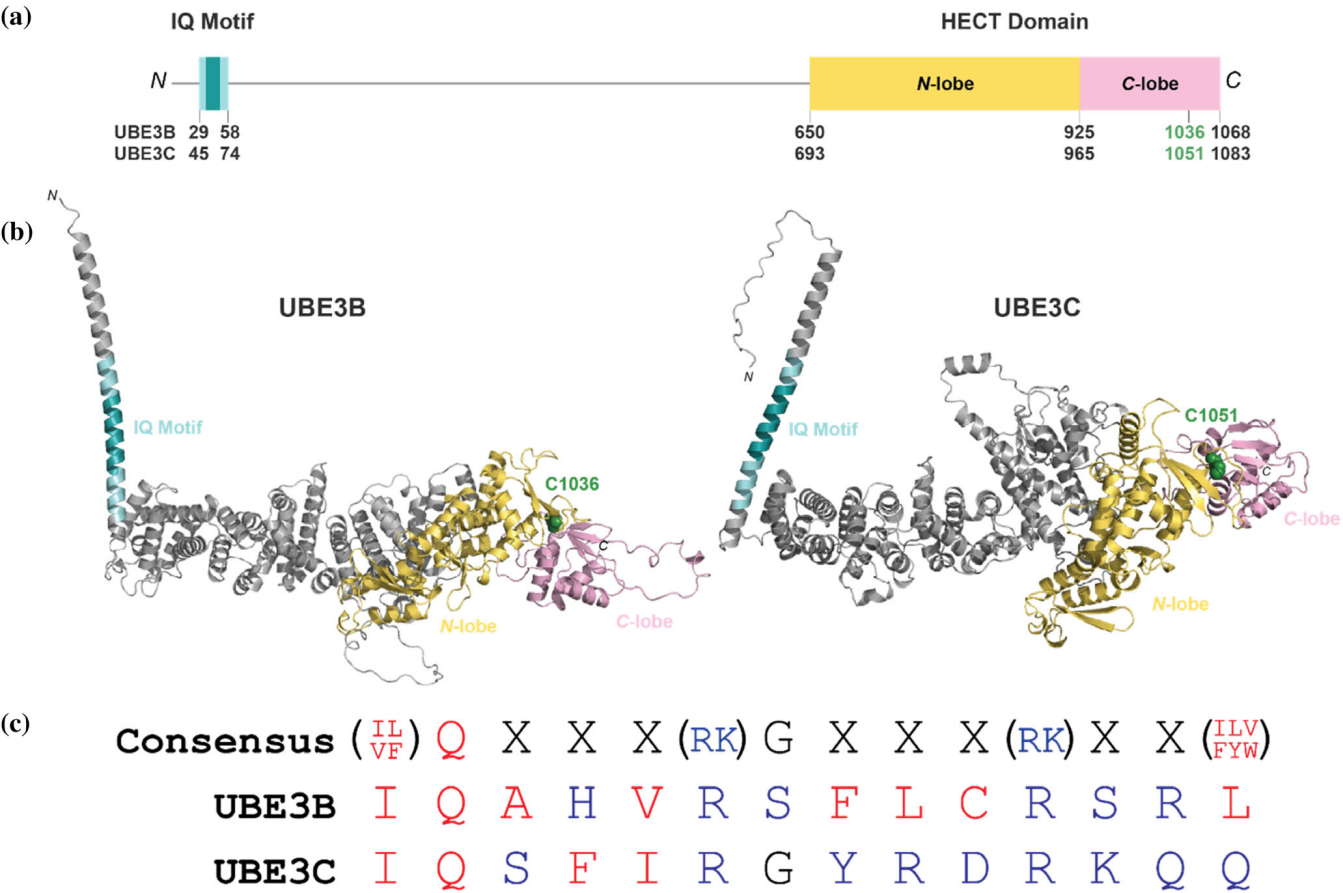
Comparison of ubiquitin protein ligase E3B (UBE3B) and ubiquitin protein ligase E3C (UBE3C) domain organization and isoleucine‐glutamine (IQ) motif sequences. (a) Domain organization of UBE3B and UBE3C highlighting the *N*‐terminal IQ motif (cyan), IQ core (teal), and *C*‐terminal homologous to E6AP carboxyl terminus (HECT) domain. The HECT *N*‐lobe is shown in yellow, and the HECT *C*‐lobe is shown in pink. (b) UBE3B and UBE3C structures retrieved from the AlphaFold Protein Structure Database, showing their overall structural similarity. The conserved catalytic cysteine within the HECT *C*‐lobe is highlighted in green. AlphaFold models: UBE3B, AF‐Q7Z3V4‐F1‐v4; UBE3C, AF‐Q15386‐F1‐v4. (c) Alignment of the UBE3B and UBE3C IQ motif cores with the IQ motif consensus sequence. Conserved hydrophobic and charged/polar residues are highlighted in red and blue, respectively.

The *N*‐terminal IQ motifs of UBE3B and UBE3C therefore provide a direct way to test this idea. IQ motifs are commonly recognized by calmodulin (CaM), a calcium‐sensing protein that regulates hundreds of cellular targets (Chin & Means, 2000; Hoeflich & Ikura, 2002). These motifs are especially relevant because they often bind CaM in the calcium‐free apo state rather than the calcium‐bound holo state (Rhoads & Friedberg, 1997). IQ motifs are defined by a 14‐residue core, generally represented as [I/L/V/F]QXXX[R/K]GXXX[R/K]XX[I/L/V/F/Y/W], but variation at nonconserved positions can strongly influence CaM‐binding affinity, lobe engagement, and complex geometry (Hoeflich & Ikura, 2002; Mruk et al., 2014). Consistent with this sequence‐dependent flexibility, experimentally determined apo‐calmodulin (apo‐CaM)/IQ complex structures reveal compact, extended, and single‐lobe recognition modes, which are summarized in Table S1. In compact complexes, both the *N*‐ and *C*‐lobes of CaM engage the IQ motif, whereas extended complexes rely more heavily on one CaM lobe, typically the *C*‐lobe (Chagot & Chazin, 2011; Feldkamp et al., 2011; Houdusse et al., 2006) (Figure 2). Thus, related IQ motifs can share canonical sequence features while still encoding different CaM‐binding behaviors.

**FIGURE 2:**
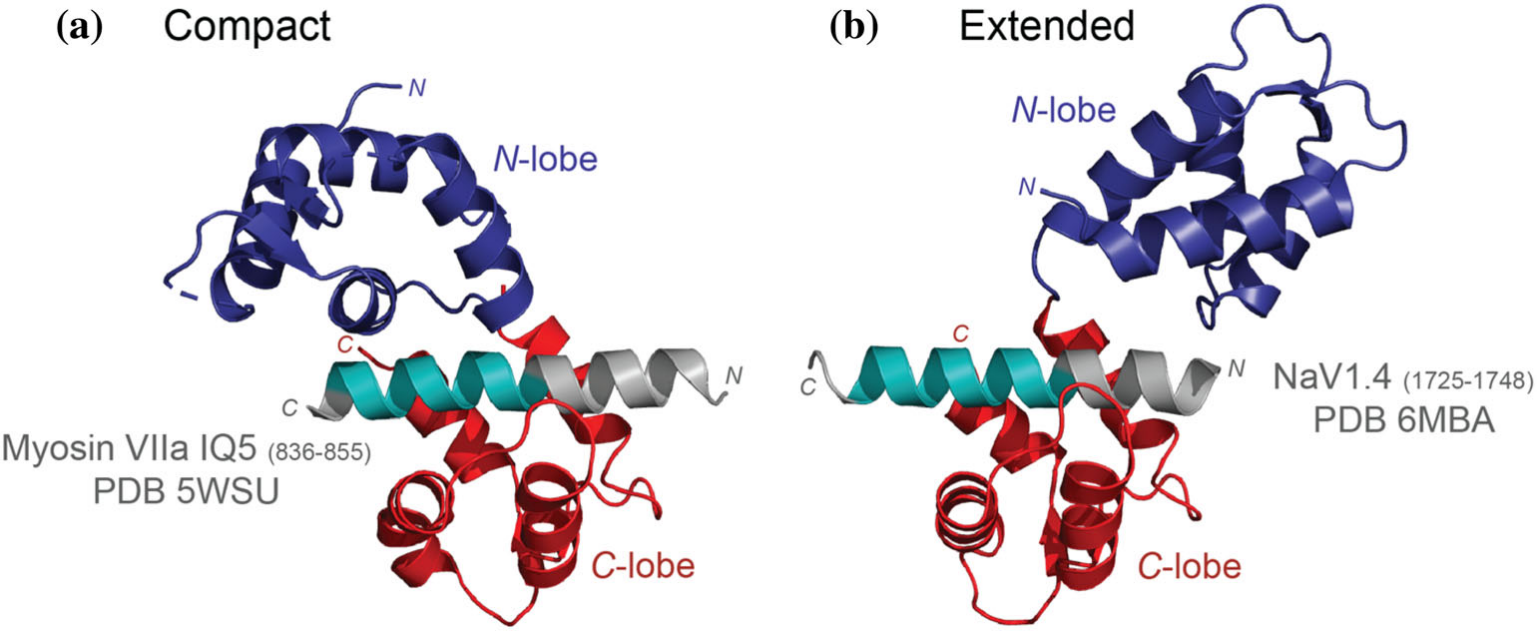
Representative compact and extended apo‐calmodulin (apo‐CaM)/isoleucine‐glutamine (IQ) motif complex conformations. Experimentally determined apo‐CaM/IQ motif structures summarized in Table S1 illustrate distinct modes of IQ motif recognition. (a) In the compact conformation, both the *N*‐lobe and *C*‐lobe of apo‐CaM engage the target IQ motif, as represented by the myosin VIIa IQ5 complex (residues 835–855; PDB 5WSU). (b) In the extended conformation, apo‐CaM adopts a more open arrangement where the target IQ motif is engaged primarily by the *C*‐lobe of CaM, as represented by the NaV1.4 complex (residues 1725–1748; PDB 6MBA). The CaM *N*‐lobe is shown in blue and the CaM *C*‐lobe in red. IQ motif‐containing regions are shown in cyan, with flanking regions shown in gray. The *N*‐ and *C*‐termini of CaM and the displayed target helices are indicated. Figures were generated using PyMOL (Schrödinger, 2024).

This sequence‐dependent flexibility makes UBE3B and UBE3C well suited for testing how conserved IQ motifs are interpreted by apo‐CaM. UBE3B was previously shown to bind CaM through its *N*‐terminal IQ motif, and this interaction was proposed to regulate UBE3B ubiquitylation activity (Braganza et al., 2017). In this model, apo‐CaM binding to the UBE3B IQ motif reduces ligase activity, while deletion of the IQ motif or increased calcium concentration relieves this inhibition (Braganza et al., 2017). UBE3C contains a homologous *N*‐terminal IQ motif, raising the possibility that CaM may regulate UBE3C through a similar mechanism. However, direct biochemical evidence for CaM recognition by the UBE3C IQ motif has remained limited, and whether the UBE3B and UBE3C IQ motifs encode conserved or divergent modes of apo‐CaM recognition is unknown.

Here, we combine isothermal titration calorimetry (ITC), nuclear magnetic resonance (NMR) spectroscopy, phylogenetic analysis, AlphaFold‐based modeling, and molecular dynamics (MD) simulations to compare how apo‐CaM recognizes the UBE3B and UBE3C IQ motifs. We show that the UBE3B IQ motif binds apo‐CaM tightly through a focused interaction surface on the CaM *C*‐lobe, whereas the UBE3C IQ motif binds weakly and produces smaller, more distributed NMR perturbations. Our structural models and MD simulations provide a framework for interpreting these experimental differences in the context of known apo‐CaM/IQ recognition modes. These findings reveal that related IQ motifs in homologous HECT ligases can be read differently by apo‐CaM, suggesting that small sequence differences can alter how CaM recognizes and potentially regulates these enzymes.

## 2 RESULTS

### 2.1 The UBE3B IQ motif preferentially engages the apo‐CaM *C* ‐lobe with high affinity

The major interaction site between apo‐CaM and the IQ motifs of UBE3B and UBE3C was first examined using ITC. ITC was used because it directly reports heat changes associated with complex formation and allows comparison of apparent binding affinity and stoichiometry under matched calcium‐free conditions. To determine whether binding preferentially involved a specific CaM lobe, titrations were performed using full‐length CaM (WT CaM), the isolated *N*‐lobe (nCaM; residues 1–81), and the isolated *C*‐lobe (cCaM; residues 76–148). Synthetic peptides corresponding to the IQ motifs of UBE3B (residues 29–58; UniProt Q7Z3V4) and UBE3C (residues 45–74; UniProt Q15386) were designed based on annotated protein sequences. These 30‐residue peptides were chosen to include the annotated IQ motif core together with flanking residues that may contribute to apo‐CaM lobe engagement and recognition specificity. Domain boundaries for the peptides were selected from the PROSITE annotation on UniProt, which included both the conserved IQ motif core and adjacent residues.

The UBE3B IQ peptide bound WT apo‐CaM with high affinity (*K*_d_ = 105 ± 18 nM) and retained substantial binding to the isolated *C*‐lobe (*K*_d_ = 270 ± 45 nM), while no measurable interaction was detected with the isolated *N*‐lobe (Figure 3a–c). The observed stoichiometry for both WT CaM and cCaM interactions was approximately 1.4, consistent with formation of a dominant IQ/CaM complex under these conditions. Though a stoichiometry of 1 has been observed in several apo‐CaM/IQ interactions, there have been reports of other stoichiometries, such as with the binding of apo‐CaM to myosin I isozymes MyoA and MyoE (Crawley et al., 2011). Together, these data indicate that the apo‐CaM *C*‐lobe serves as the primary interaction surface for the UBE3B IQ motif, consistent with previously characterized apo‐CaM/IQ complexes.

**FIGURE 3:**
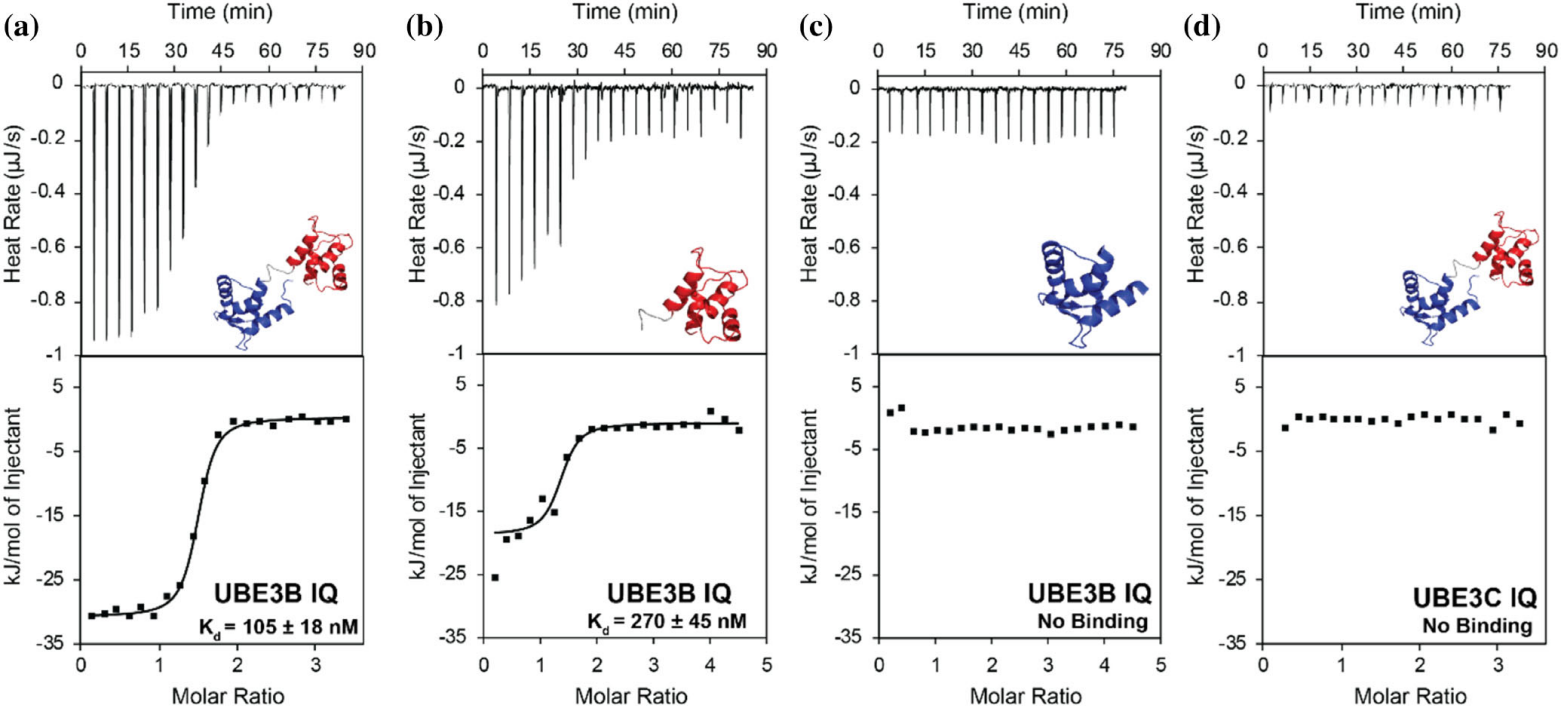
The ubiquitin protein ligase E3B (UBE3B) isoleucine‐glutamine (IQ) motif preferentially binds the apo‐calmodulin (apo‐CaM) *C*‐lobe. Isothermal titration calorimetry experiments were performed by titrating a synthetic UBE3B IQ peptide (residues 29–58) into full‐length apo‐CaM, the isolated apo‐CaM *N*‐lobe (residues 1–81), or the isolated apo‐CaM *C*‐lobe (residues 76–148) under calcium‐free conditions. Representative thermograms and binding isotherms are shown for UBE3B IQ binding to (a) full‐length CaM, (b) the isolated CaM *C*‐lobe, and (c) the isolated CaM *N*‐lobe. UBE3B IQ bound full‐length apo‐CaM with high affinity and retained binding to the isolated *C*‐lobe, whereas no measurable binding was detected with the isolated *N*‐lobe. (d) Synthetic ubiquitin protein ligase E3C (UBE3C) IQ peptide, corresponding to residues 45–74 of UBE3C, was titrated into full‐length apo‐CaM under identical conditions and produced small heat changes that could not be reliably fit or unambiguously distinguished from potential peptide dilution effects. Each experiment was performed in triplicate.

In contrast, titration of the UBE3C IQ peptide into WT apo‐CaM produced substantially smaller heat changes that could not be reliably fit under identical experimental conditions (Figure 3d). Because peptide‐into‐buffer control titrations were not performed, these small heat changes cannot be unambiguously distinguished from contributions arising from peptide dilution. We therefore did not use the UBE3C ITC data to estimate a binding affinity. Subsequent NMR titrations independently supported a weaker interaction between the UBE3C IQ motif and apo‐CaM.

### 2.2 NMR titrations reveal distinct apo‐CaM recognition modes for the UBE3B and UBE3C IQ motifs

To further characterize apo‐CaM recognition by the UBE3B and UBE3C IQ motifs, we performed NMR titration experiments using increasing concentrations of synthetic IQ peptides added to isotopically labeled apo‐CaM. Overlayed ^1^H‐^15^N heteronuclear single quantum coherence (HSQC) spectra revealed distinct modes of apo‐CaM recognition for the two complexes, consistent with different exchange regimes (Figures 4 and 5).

**FIGURE 4:**
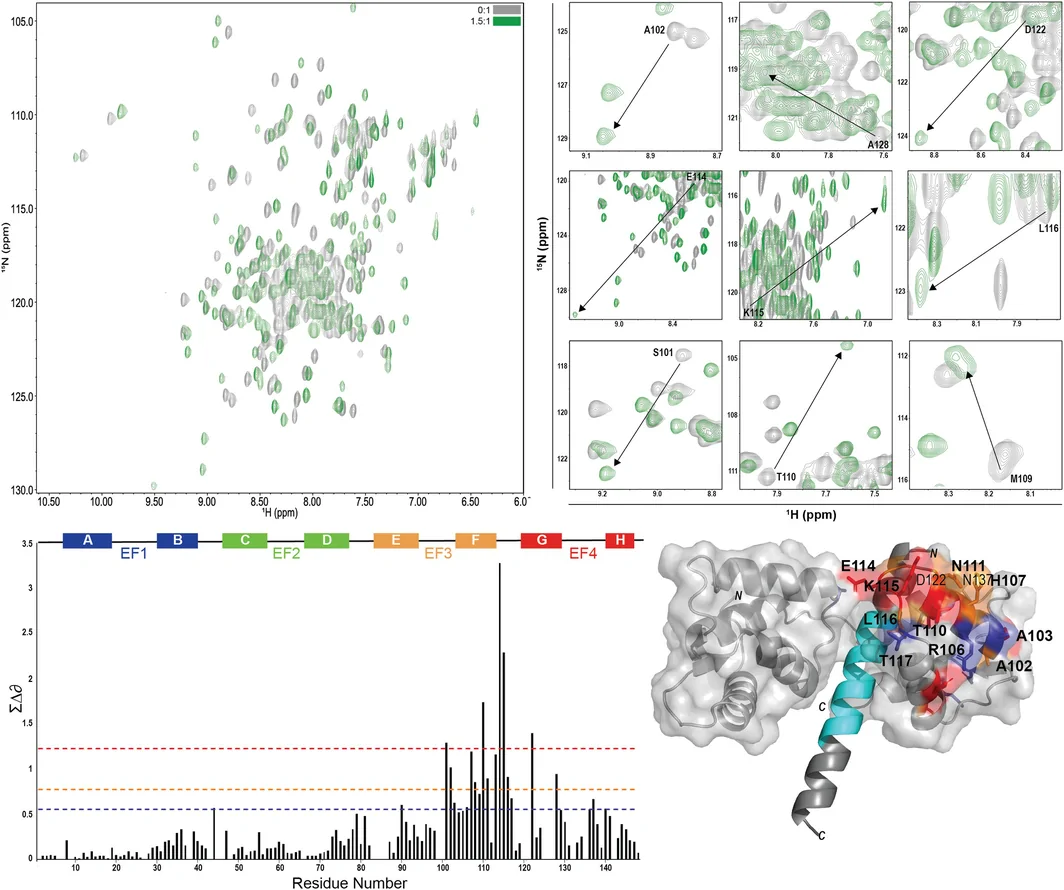
Ubiquitin protein ligase E3B (UBE3B) isoleucine‐glutamine (IQ) binding localizes to the apo‐calmodulin (apo‐CaM) *C*‐lobe. ^1^H‐^15^N heteronuclear single quantum coherence spectra of apo‐CaM alone (gray) and apo‐CaM saturated with UBE3B IQ peptide at a 1.5:1 peptide: Protein ratio (green) are overlaid to show residue‐specific peak movement upon complex formation. Insets highlight nine residues with the largest chemical shift perturbations (CSPs) observed. CSP magnitudes were calculated for each residue using ΣΔδ = |Δδ^1^H| + 0.2|Δδ^15^N| and plotted as a histogram. Residues exceeding the mean plus 0.5, 1, or 2 standard deviations are colored blue, orange, or red, respectively. Residues with significant perturbations were mapped onto the AlphaFold3/MD‐derived apo‐CaM/UBE3B IQ model using the same color scheme. EF‐hands 1–4 (EF1–EF4) are indicated on the apo‐CaM structure for reference. The largest perturbations were concentrated within the apo‐CaM *C*‐lobe, indicating that the UBE3B IQ motif primarily binds this region of CaM.

**FIGURE 5:**
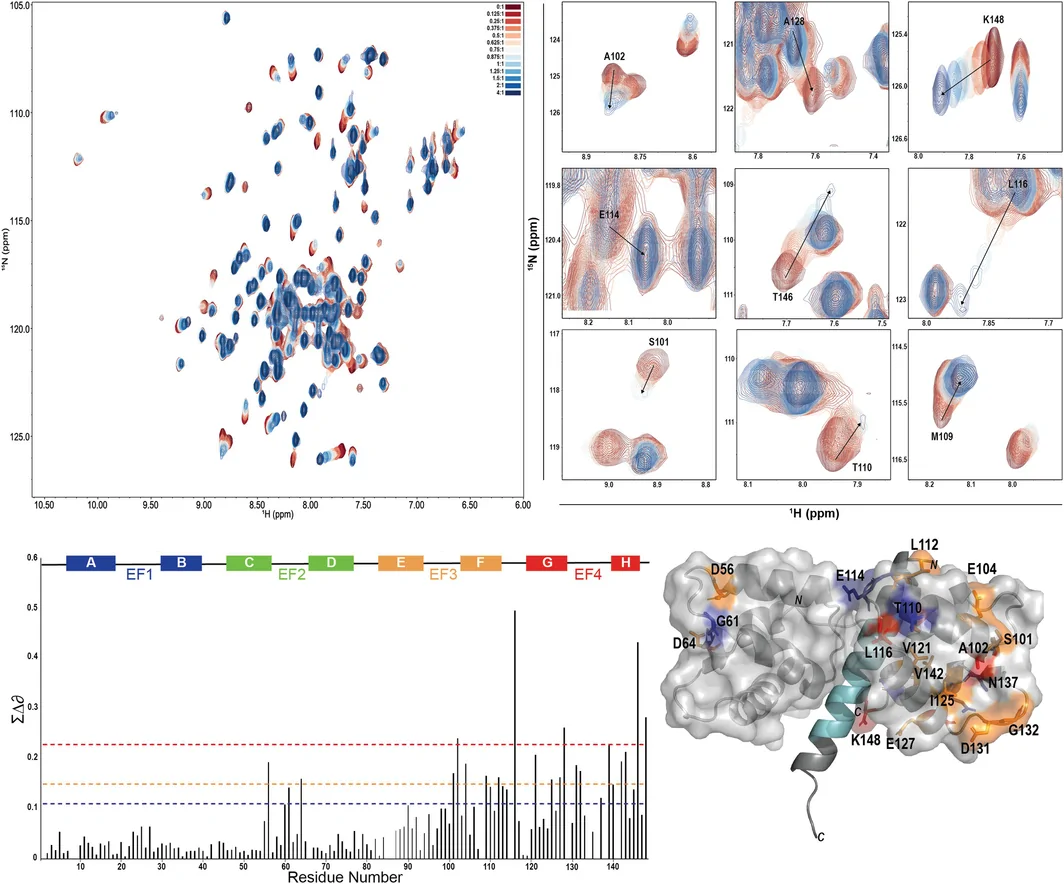
Ubiquitin protein ligase E3C (UBE3C) IQ binding produces weaker and more distributed perturbations in apo‐calmodulin (apo‐CaM). ^1^H‐^15^N heteronuclear single quantum coherence spectra were collected as apo‐CaM (red) was titrated with increasing concentrations of UBE3C IQ peptide, up to a 4:1 peptide: Protein ratio (blue). Spectra are shown as an overlay from apo‐CaM alone to increasing UBE3C IQ concentrations, with selected residues highlighted to show gradual peak movement during titration. Insets show nine residues with the largest chemical shift perturbations (CSPs) observed. CSP magnitudes were calculated for each residue using the weighted additive equation ΣΔδ = |Δδ^1^H| + 0.2|Δδ^15^N| and plotted as a histogram. Residues exceeding the mean plus 0.5, 1, or 2 standard deviations are colored blue, orange, or red, respectively. Residues with significant perturbations were mapped onto the AlphaFold3/MD‐derived apo‐CaM/UBE3C IQ model using the same color scheme. EF‐hands 1–4 (EF1–EF4) are indicated on the apo‐CaM structure for reference. Compared with ubiquitin protein ligase E3B IQ, the UBE3C IQ motif produced smaller perturbations that were distributed more broadly across apo‐CaM, consistent with a weaker and less localized binding interaction.

Addition of the UBE3B IQ peptide caused peaks from free apo‐CaM to disappear while new peaks for the bound complex appeared, indicating slow exchange on the NMR timescale (Figure 4). Because the NMR titration was performed at 300 μM apo‐CaM, substantially above the submicromolar affinity measured independently by ITC, the experiment was in the tight‐binding regime and was not sufficiently sensitive to determine an independent quantitative *K*_d_. In contrast, addition of the UBE3C IQ peptide caused apo‐CaM peaks to shift gradually during the titration, consistent with fast exchange and weaker binding (Figure 5). Global fitting of the concentration‐dependent chemical‐shift trajectories of five well‐resolved resonances (G113, L116, D131, A147, and K148) to a 1:1 finite‐concentration binding model yielded a *K*_d_ of 180 ± 20 μM. Even at the highest peptide concentrations tested, several resonances continued to shift, indicating a substantially weaker interaction.

To map the interaction surfaces involved in each complex, backbone assignments of the saturated apo‐CaM/UBE3B complex were determined in‐house using standard triple‐resonance NMR experiments and used to calculate chemical shift perturbations (CSPs) relative to apo‐CaM. Mapping these perturbations onto AlphaFold3 (AF3)‐derived structural models showed that the UBE3B IQ motif primarily affected residues within the apo‐CaM *C*‐lobe, particularly across the I100–I130 region (Figure 4). The largest perturbations were observed for residues S101, T110, E114, K115, and D122, which collectively formed a localized interaction surface within the *C*‐lobe of apo‐CaM.

Consistent with its weaker affinity, the UBE3C IQ motif produced substantially smaller and more distributed CSPs than UBE3B (Figure 5). The largest CSP observed for the UBE3B complex was 3.28 ppm at residue E114, whereas the largest perturbation observed for the UBE3C complex reached only 0.495 ppm at residue L116. Despite partial overlap in perturbed residues between the two complexes, mapping of the UBE3C perturbations revealed a broader and less localized interaction surface compared with the focused *C*‐lobe interaction surface observed for UBE3B (Figure 5). Taken together, these data show that the UBE3B IQ motif binds apo‐CaM more tightly and at a more defined site, whereas the UBE3C IQ motif interacts more weakly and produces smaller, more distributed perturbations consistent with a less well‐defined interaction.

### 2.3 Structural modeling and MD simulations provide a framework for divergent apo‐CaM engagement mechanisms

To better understand the structural basis for the different apo‐CaM binding behaviors observed experimentally, we generated AF3 models of the UBE3B and UBE3C IQ/apo‐CaM complexes and analyzed them using multimicrosecond MD simulations. These models were used as structural hypotheses to interpret the ITC and NMR data, rather than as experimentally determined complex structures.

Both predicted complexes adopted compact apo‐CaM conformations resembling previously characterized IQ/apo‐CaM structures, with the apo‐CaM *C*‐lobe engaging the *N*‐terminal region of the IQ helix (Figure 6a). The overall architectures of the UBE3B and UBE3C complexes were highly similar, with a backbone root mean squared deviation (RMSD) of 1.2 Å between the predicted structures after excluding terminal residues (Figure 6a, right panel). In both complexes, conserved hydrophobic IQ residues inserted into the semi‐open hydrophobic cleft of the apo‐CaM *C*‐lobe, while the apo‐CaM *N*‐lobe contacted the *C*‐terminal portion of the IQ helix. Specifically, the *C*‐lobe gripped the *N*‐terminal IQ region centered on the I8Q9xxxR13 segment, with Q9 and R13 positioned near linker 3 of the *C*‐lobe and the side chains of I8, V/I12, and F/Y15 inserted into the *C*‐lobe hydrophobic cleft. The *N*‐lobe contacted the *C*‐terminal region of the motif, including the S/G14xxxR18 region and residues near L/Q21, through surfaces formed by CaM helices A and B and linker 1 (Figure 6a,b). These interface features are consistent with contact patterns observed in previously solved apo‐CaM/IQ complexes, particularly compact recognition modes involving both CaM lobes (Figure 6c; Table S1).

**FIGURE 6:**
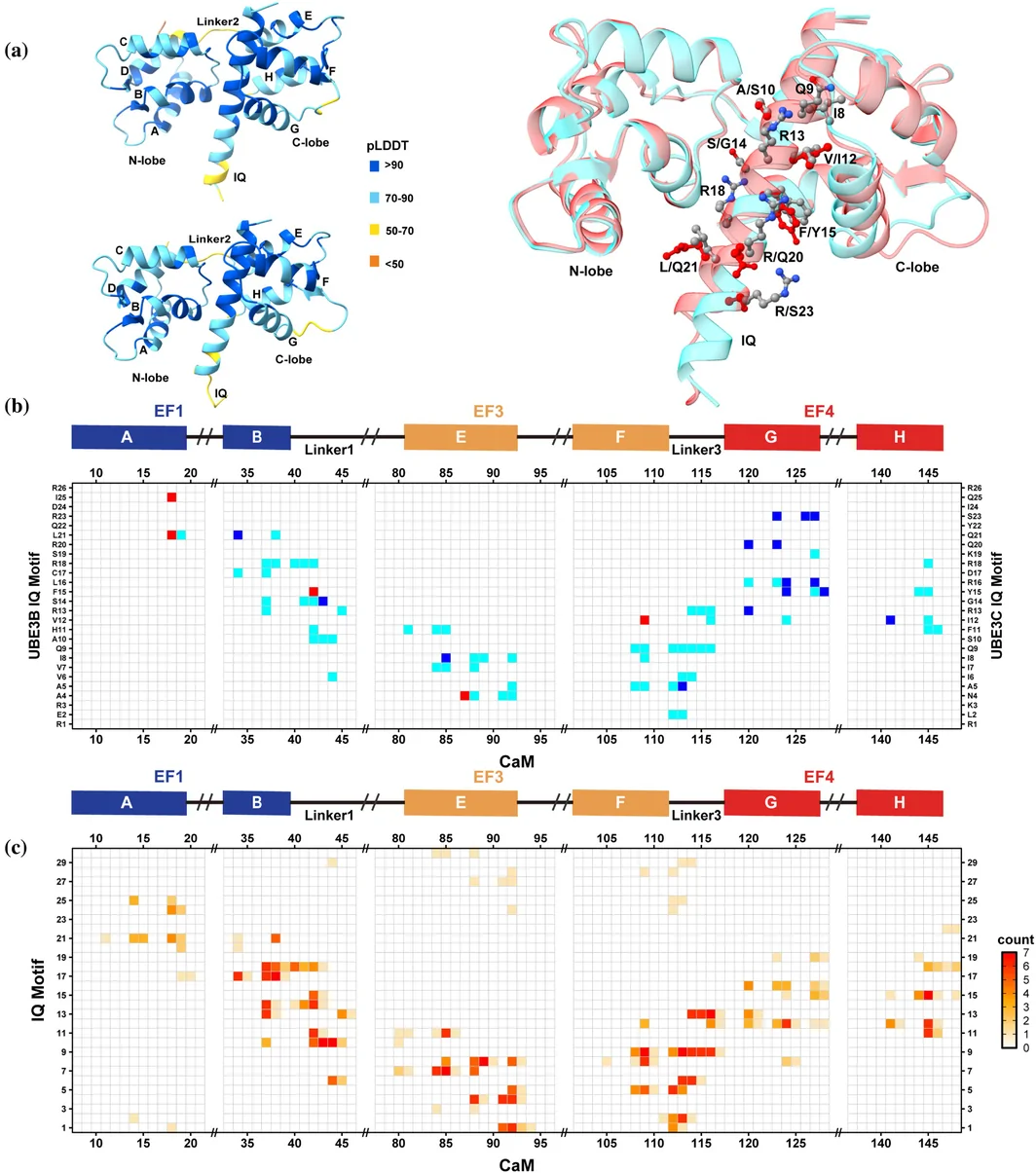
AlphaFold3 (AF3) models predict similar compact apo‐calmodulin (apo‐CaM)/IQ architectures for ubiquitin protein ligase E3B (UBE3B) and ubiquitin protein ligase E3C (UBE3C). (a) AF3‐predicted structures of the UBE3B IQ/apo‐CaM complex and the UBE3C IQ/apo‐CaM complex are shown with pLDDT confidence scores color‐coded. CaM is composed of an *N*‐lobe containing EF‐hands 1 and 2 and a *C*‐lobe containing EF‐hands 3 and 4, with α‐helices labeled A–H. The linker regions connecting the EF‐hands are also indicated. The predicted UBE3B (cyan) and UBE3C (red) complexes were superimposed to compare their overall architectures, yielding a backbone RMSD of 1.2 Å after exclusion of terminal residues. The terminal five residues of each IQ motif and the terminal five residues of CaM were excluded from the superposition. IQ consensus residues I8, Q9, R13, and R18, along with additional residues contributing major contacts at the IQ/apo‐CaM interface, are shown as sticks. (b) Contact map of the predicted IQ/apo‐CaM interfaces. Contacts shared by both complexes are shown in cyan, contacts unique to the UBE3B complex are shown in blue, and contacts unique to the UBE3C complex are shown in red. (c) Contact map of previously solved apo‐CaM/IQ structures used for comparison. The map was generated from seven IQ/apo‐CaM‐containing proteins, representing nine total interfaces: PDB IDs 2IX7 (Houdusse et al., 2006), 4BYF (Feldkamp et al., 2011), 4LZX (Chagot & Chazin, 2011), 4R8G (Lu et al., 2015), 5I0I (Ropars et al., 2016), 5V7X (Mentes et al., 2018), and 5WSU (Li et al., 2017). The IQ motif consensus sequence is shown as [I/L/V]^8^Q^9^xxxR^13^[G/x]^14^xxx[R/K]^18^, and CaM EF‐hand regions are indicated above the contact maps.

Despite these similarities, our MD simulations revealed substantial differences in conformational stability and interface organization between the two complexes. Across three independent simulations for each complex, totaling 12 μs of aggregate simulation time, both complexes retained their overall architectures, but the UBE3C complex deviated more strongly from its starting model in both CaM lobes and in the IQ motif (Figure 7a,b). In the UBE3C simulations, up to nine residues near the *C*‐terminus of the IQ peptide lost helical structure and sampled conformations oriented toward the CaM *N*‐lobe (Figure 7b). The apo‐CaM linker 3 region and helix G also exhibited greater fluctuations in the UBE3C complex than in the UBE3B complex (Figure 7c,d). In contrast, the UBE3B complex retained a more stable *C*‐lobe‐centered interface throughout the simulations.

**FIGURE 7:**
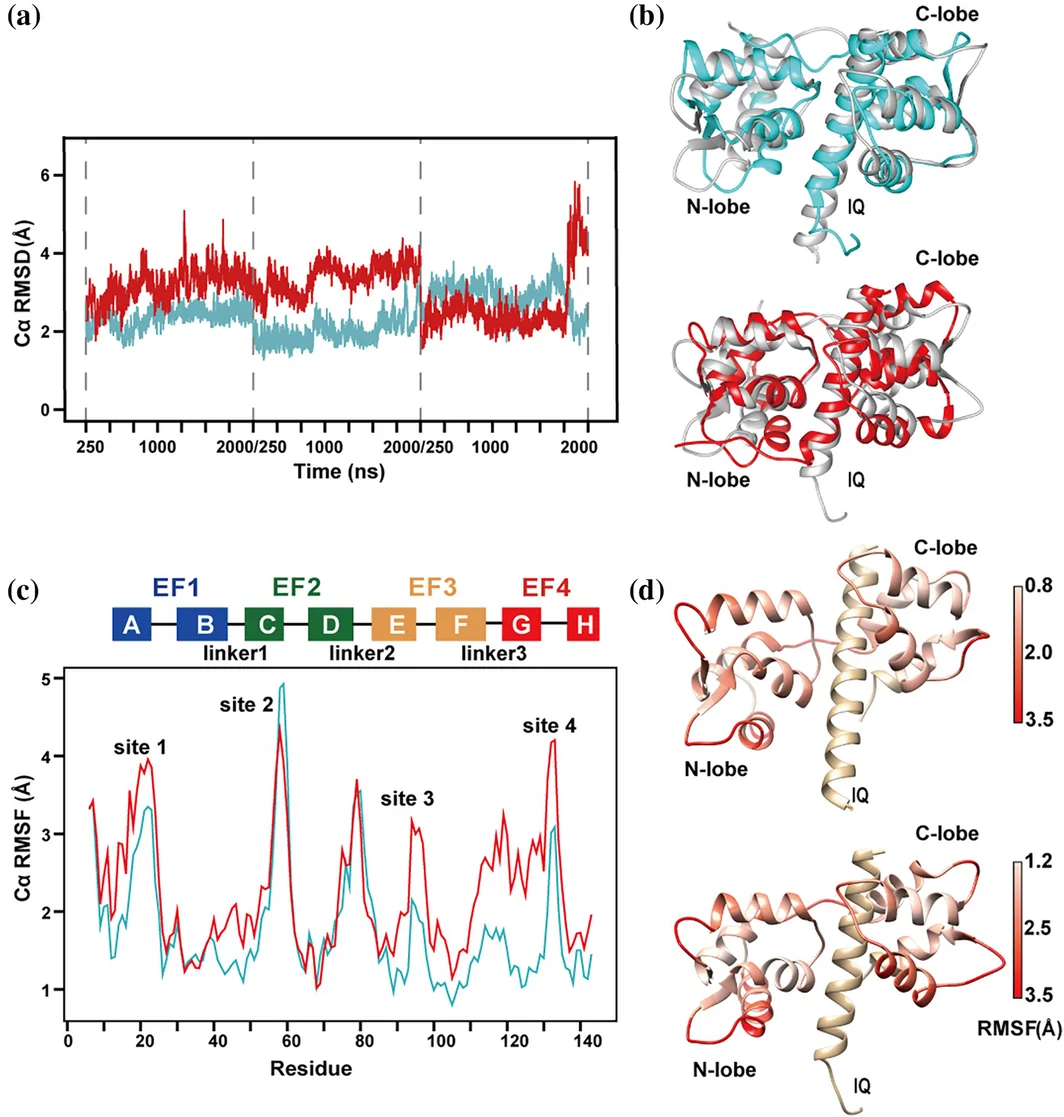
Root‐mean‐square deviation (RMSD) and fluctuation analyses of ubiquitin protein ligase E3B (UBE3B) and ubiquitin protein ligase E3C (UBE3C) IQ/apo‐calmodulin (apo‐CaM) complexes. (a) Cα RMSD of the UBE3B IQ/apo‐CaM complex (cyan) and UBE3C IQ/apo‐CaM complex (red) relative to their minimized starting structures across three independent MD simulations. The first 250 ns of each trajectory were excluded from analysis. *N*‐ and *C*‐terminal residues of CaM and the IQ motifs were excluded as described in section 4. (b) Representative simulation snapshots of the UBE3B and UBE3C complexes superimposed on their minimized starting structures shown in gray. (c) Cα root‐mean‐square fluctuation (RMSF) of apo‐CaM in the UBE3B complex (cyan) and UBE3C complex (red). CaM EF‐hand motifs and Ca^2+^‐binding sites are indicated. (d) RMSF values mapped onto representative structures of the UBE3B IQ/apo‐CaM complex (top) and UBE3C IQ/apo‐CaM complex (bottom), highlighting regions of increased flexibility during the simulations. The IQ motif consensus is highlighted in yellow.

Radius of gyration analysis further supported broader conformational sampling by the UBE3C complex. The Rg values of apo‐CaM and of the individual CaM lobes were lower in both IQ‐bound complexes than previously reported for apo‐CaM alone, consistent with compaction upon IQ peptide binding (Figure 8a). However, the UBE3C complex displayed broader Rg distributions than the UBE3B complex, suggesting greater conformational heterogeneity. Buried surface area (BSA) analysis revealed a similar pattern at the IQ/apo‐CaM interface (Figure 8b). The UBE3B complex sampled a comparatively focused interface distribution, whereas the UBE3C complex showed a broader and bimodal distribution. The major UBE3C subgroup had a median BSA similar to that of the UBE3B complex, whereas a minor subgroup showed a larger BSA due to additional contacts between the partially unfolded *C*‐terminal region of the UBE3C IQ peptide and the CaM *N*‐lobe (Figure 8c,d). Because these additional contacts were not broadly persistent, we interpret them as evidence of increased conformational sampling rather than as a defined secondary binding mode.

**FIGURE 8:**
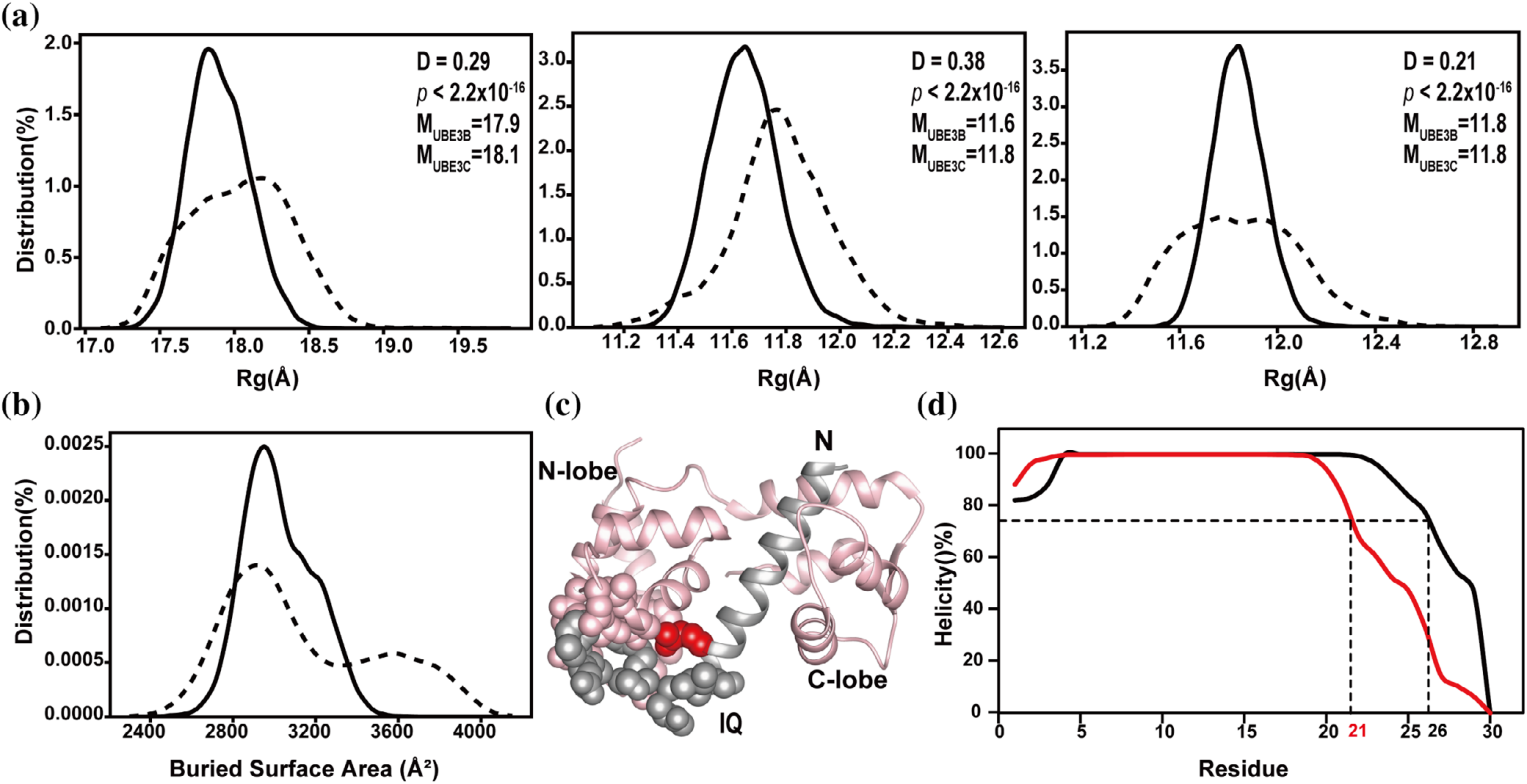
Molecular dynamics (MD) simulations show broader conformational sampling by the ubiquitin protein ligase E3C (UBE3C) IQ/apo‐calmodulin (apo‐CaM) complex. (a) Radius of gyration (Rg) distributions calculated from MD simulations for apo‐CaM overall, excluding the five *N*‐ and *C*‐terminal residues, the apo‐CaM *N*‐lobe (residues 6–75), and the apo‐CaM *C*‐lobe (residues 82–143). Solid and dashed lines represent the UBE3B and UBE3C complexes, respectively. Kolmogorov‐Smirnov statistics are shown in each panel, where D indicates the maximum difference between distributions and M indicates the median. (b) Distribution of buried surface area at the IQ/apo‐CaM interface for the UBE3B and UBE3C complexes. The UBE3C complex samples a broader and more heterogeneous interface distribution than UBE3B, indicating that the UBE3C IQ motif engages apo‐CaM through a more variable binding surface during the simulations. (c) Representative snapshot from the larger buried‐surface‐area subgroup of the UBE3C complex. CaM is shown in pink and the IQ motif in gray. The *C*‐terminal region of the UBE3C IQ motif contacts the EF‐hand 1 of CaM. Interacting regions are shown as spheres, with Q21 highlighted in red. (d) Helical content of each IQ motif residue during MD simulations. UBE3B and UBE3C are shown in black and red, respectively. The dashed horizontal line marks the 75% helicity threshold. Compared with UBE3B, the UBE3C IQ motif shows reduced helicity near its *C*‐terminus, consistent with increased conformational flexibility.

Analysis of persistent interface contacts identified sequence features that may stabilize the UBE3B complex more effectively than the UBE3C complex (Figure 9a,b). In the UBE3B model and simulations, residues outside the canonical IQ consensus, including R20 and R23, formed persistent charge‐based contacts with residues in or near CaM helix G, while F15 and L16 supported hydrophobic packing against the CaM *C*‐lobe. In UBE3C, the corresponding Y15 and R16 substitutions altered this local packing environment and were associated with a less compact interface in the simulations. Consistent with this interpretation, the distance between the IQ helix and apo‐CaM helix G was broader and more variable in the UBE3C complex than in the UBE3B complex (Figure 9c).

**FIGURE 9:**
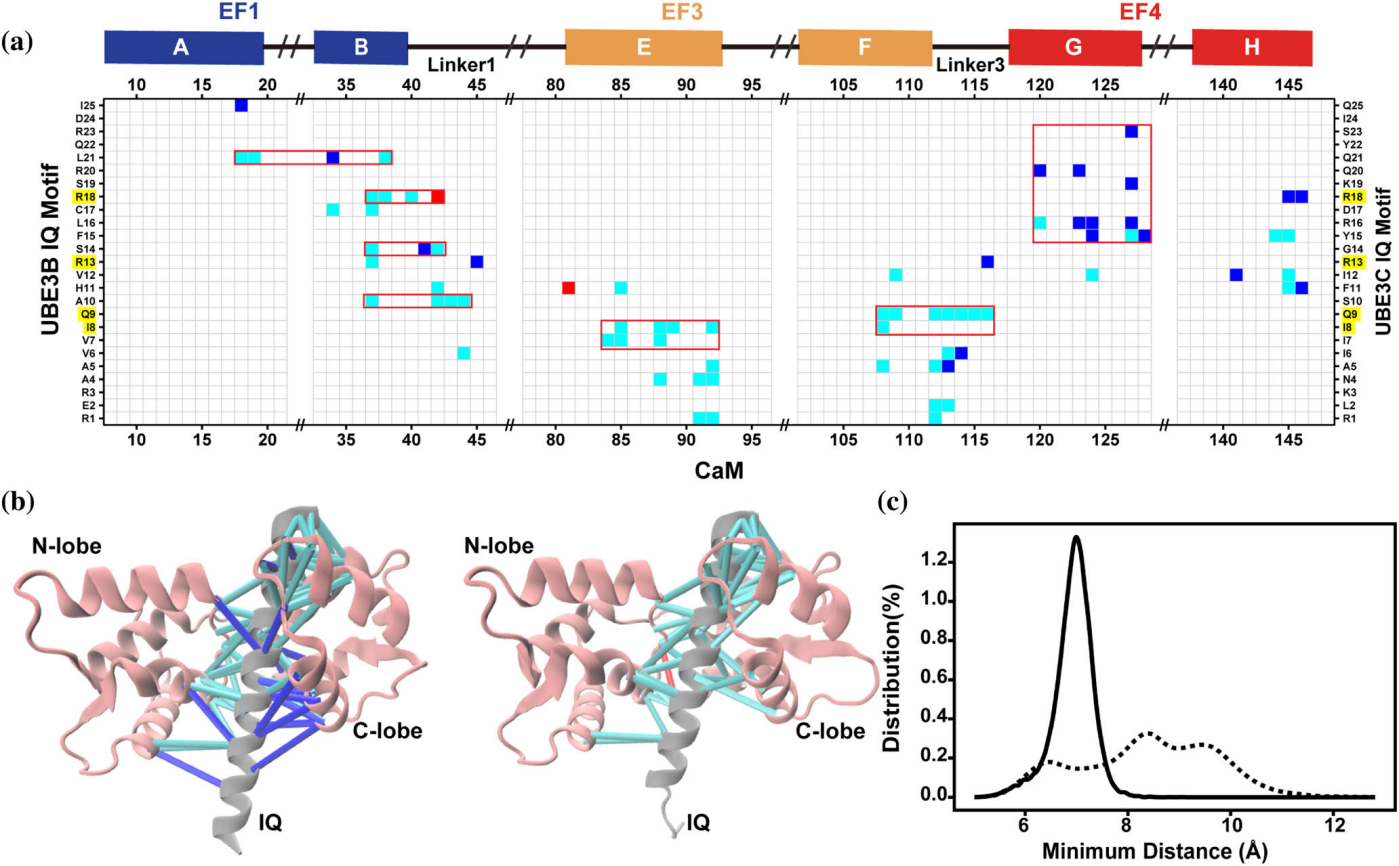
Molecular dynamics (MD) simulations identify distinct apo‐calmodulin (apo‐CaM) interface contacts in the ubiquitin protein ligase E3B (UBE3B) and ubiquitin protein ligase E3C (UBE3C) IQ complexes. (a) A contact map of IQ/apo‐CaM interactions observed during MD simulations. Contacts shared by both complexes are shown in cyan, contacts unique to the UBE3B complex are shown in blue, and contacts unique to the UBE3C complex are shown in red. Consensus IQ motif residues are highlighted in yellow. UBE3B forms additional contacts with apo‐CaM beyond the shared interface. The red open boxes highlight the prominent interaction regions. (b) Structural view of persistent IQ/apo‐CaM interface contacts. CaM is shown in pink and the IQ motif in gray. Shared contacts are shown as cyan sticks, while contacts unique to the UBE3B complex (left panel) and UBE3C complex (right panel) are shown as blue and red sticks, respectively. UBE3B forms additional contacts near the apo‐CaM G helix, whereas the UBE3C interface is less tightly packed. (c) Distribution of interhelical distances between apo‐CaM helix G (D118–E127) and the IQ motif region spanning R/K3–L/Q21. Solid lines represent the UBE3B complex, and dashed lines represent the UBE3C complex. The minimized models showed an initial interhelical distance of 6.7 Å for UBE3B and 8.1 Å for UBE3C. During simulation, this distance remained more variable in the UBE3C complex, indicating a less compact interaction with the apo‐CaM *C*‐lobe.

Together, these simulations provide a structural framework for interpreting the distinct apo‐CaM binding behaviors observed experimentally. The UBE3B IQ motif forms a more compact and persistent *C*‐lobe‐centered interface in the models and simulations, consistent with its tighter binding and slow‐exchange behavior by NMR. In contrast, the UBE3C IQ motif shows weaker binding experimentally and samples a more heterogeneous interface computationally, consistent with a less well‐defined apo‐CaM interaction.

### 2.4 UBE3B and UBE3C IQ motifs follow divergent evolutionary trajectories

Although the UBE3B and UBE3C IQ motifs retain canonical sequence features, their markedly different apo‐CaM binding affinities, NMR exchange behaviors, and interaction surfaces suggest that these motifs have not remained functionally equivalent. Instead, they may have diverged evolutionarily to support distinct modes of CaM recognition and regulation. To examine this possibility, we performed phylogenetic analysis using approximately 450 IQ motif‐containing proteins.

Our analysis revealed that the IQ motifs of UBE3B and UBE3C occupy distinct evolutionary branches and display stronger relationships to different groups of IQ‐containing proteins (Figure S1). The UBE3B IQ motif shares features with myosin‐associated apo‐CaM/IQ complexes, including preservation of a hydrophobic interaction surface that supports compact *C*‐lobe binding (Houdusse et al., 2006). In contrast, the UBE3C IQ motif contains substitutions that disrupt this hydrophobic surface and produce a weaker, more distributed interaction pattern reminiscent of the broader recognition modes observed among ion channel‐associated IQ motifs (Chagot & Chazin, 2011; Feldkamp et al., 2011). Together with our biochemical, NMR, and simulation data, these findings show that IQ motifs with similar consensus features can nevertheless engage apo‐CaM through markedly different recognition mechanisms driven by local sequence context.

## 3 DISCUSSION

In this study, we show that the conserved IQ motifs of the related HECT E3 ubiquitin ligases UBE3B and UBE3C engage apo‐CaM through markedly different molecular recognition mechanisms. Although both motifs contain canonical IQ sequence features and adopt similar overall architectures in AF3 models, their biochemical, NMR, and MD data exhibit distinct interaction behaviors. The UBE3B IQ motif forms a compact and stabilized interaction centered on the apo‐CaM *C*‐lobe, whereas the UBE3C IQ motif engages apo‐CaM weakly through a broader and more heterogeneous interaction surface. Together, these findings demonstrate that conserved IQ motifs can encode distinct modes of apo‐CaM recognition through relatively subtle changes in local sequence context.

A limitation of the present study is that the UBE3B and UBE3C apo‐CaM/IQ complexes were not determined experimentally by X‐ray crystallography, NMR structure calculation, or cryo‐EM. The structural models should therefore be interpreted as experimentally informed hypotheses rather than definitive complex structures. The strongest direct experimental observations are the ITC binding behavior, the different NMR exchange regimes, and the distinct CSP patterns observed for apo‐CaM upon addition of the UBE3B and UBE3C IQ peptides. The AF3 models and MD simulations provide a useful framework for interpreting these observations in the context of known apo‐CaM/IQ recognition modes, but they do not by themselves establish a unique binding register or exclude alternative conformations.

Several alternative explanations for the weaker UBE3C response should also be considered. The smaller UBE3C‐induced CSPs could reflect weaker affinity, faster exchange, increased conformational averaging, multiple peptide registers, or reduced specificity of the isolated peptide relative to the full‐length protein context. Similarly, the nonideal apparent stoichiometries observed in the ITC and NMR titrations may reflect concentration uncertainty, partial peptide inactivity, or limitations of fitting weak or heterogeneous interactions to a simple one‐site model, rather than a strict higher‐order binding mechanism. Future studies using targeted mutagenesis, orthogonal binding assays, or full‐length UBE3B and UBE3C proteins will be important for testing the specific interface features proposed here and for determining how apo‐CaM binding influences ligase activity in a cellular context.

The distinct apo‐CaM recognition behaviors of the UBE3B and UBE3C IQ motifs appear to arise from differences in local binding organization rather than large‐scale structural rearrangements. AF3 modeling predicted broadly similar compact apo‐CaM/IQ architectures for both complexes, with the CaM *C*‐lobe engaging the *N*‐terminal region of the IQ helix, consistent with previously characterized apo‐CaM/IQ structures (Chagot & Chazin, 2011; Houdusse et al., 2006). However, NMR and MD simulations revealed substantial differences in the stability and organization of these interactions. The UBE3B IQ motif produced localized *C*‐lobe CSPs, slow exchange behavior, and stable hydrophobic packing interactions during simulation, all consistent with the formation of a compact and well‐defined apo‐CaM complex. In contrast, the UBE3C IQ motif produced weaker and more broadly distributed perturbations together with fast exchange behavior and increased conformational heterogeneity during MD simulations. These differences appear to arise in part from substitutions near the hydrophobic CaM‐binding surface, where the UBE3B residues F15 and L16 preserve hydrophobic packing interactions commonly observed in compact apo‐CaM/IQ complexes (Houdusse et al., 2006), whereas the corresponding UBE3C residues Y15 and R16 disrupt this interaction surface. Together, these findings suggest that relatively subtle sequence changes outside the canonical IQ consensus residues can substantially reshape apo‐CaM binding behavior, complex stability, and recognition dynamics (Feldkamp et al., 2011; Mruk et al., 2014).

The divergent apo‐CaM binding responses of the UBE3B and UBE3C IQ motifs further illustrate the structural and functional versatility of IQ/CaM interactions. Previous structural studies have shown that apo‐CaM can engage IQ motifs through multiple recognition modes, including compact complexes in which both CaM lobes contact the IQ helix (Houdusse et al., 2006; Lu et al., 2015; Ropars et al., 2016) and more extended complexes that rely more heavily on a single lobe (Chagot & Chazin, 2011; Feldkamp et al., 2011). The UBE3B IQ motif shares several features with compact myosin‐associated apo‐CaM/IQ complexes, including preservation of a hydrophobic interaction surface that packs against the CaM *C*‐lobe (Houdusse et al., 2006). In contrast, the UBE3C IQ motif disrupts this hydrophobic surface through polar and charged substitutions near the *C*‐lobe binding region, resulting in weaker and more distributed apo‐CaM engagement. Together, these findings illustrate how IQ motifs can retain a recognizable consensus sequence while substantially tuning CaM recognition through local sequence variation.

More broadly, these findings support the emerging view that regulatory specificity within the HECT E3 ubiquitin ligase family is frequently encoded outside the conserved catalytic domain. Recent cryo‐EM studies have shown that individual HECT E3 ligases are organized in different ways to control ubiquitin transfer, oligomerization, substrate recognition, and catalytic activity (Hodakova et al., 2023; Huber et al., 2026; Wang et al., 2023). In this context, the different apo‐CaM binding modes observed for UBE3B and UBE3C suggest that CaM‐mediated regulation may have evolved differently among HECT E3 ligases. Although both ligases contain an *N*‐terminal IQ motif, our results demonstrate that these motifs bind to apo‐CaM in very different ways, which may allow UBE3B and UBE3C to respond differently to changes in cellular Ca^2+^ levels. More generally, these findings suggest that similar short sequence motifs do not always produce the same regulatory outcome, especially in large, multidomain signaling proteins such as the HECT E3 ligases.

Several important questions remain regarding how apo‐CaM recognition influences the biological functions of UBE3B and UBE3C in cells. Previous work suggested that apo‐CaM binding suppresses UBE3B ubiquitin ligase activity in a Ca^2+^‐dependent manner (Braganza et al., 2017). In additional ITC experiments conducted in our laboratory (data not shown), Ca^2+^‐saturated CaM produced substantially smaller heat changes upon titration with the UBE3B IQ peptide than were observed under Ca^2+^‐free conditions, and the data could not be reliably fit to obtain a binding affinity. These results are consistent with markedly weakened CaM recognition of the UBE3B IQ motif upon Ca^2+^ saturation. Whether UBE3C similarly exhibits Ca^2+^‐dependent modulation of CaM binding remains to be determined. This raises the possibility that the weaker and more conformationally variable apo‐CaM interaction observed for UBE3C supports a distinct regulatory response. The divergent apo‐CaM recognition modes may also reflect the distinct biological contexts in which UBE3B and UBE3C function. UBE3B is strongly associated with neuronal development and neurodevelopmental disease (Basel‐Vanagaite et al., 2012; 2014; Braganza et al., 2017; Cheon et al., 2019; Flex et al., 2013; Yilmaz et al., 2018), whereas UBE3C appears to function more broadly in proteostasis, protein quality control, autophagy, and cancer‐associated pathways (Cui et al., 2024; Sun et al., 2024; You & Pickart, 2001). Future studies using full‐length proteins will therefore be important for defining how apo‐CaM binding to UBE3B and UBE3C influences ligase activity, substrate recognition, and cellular signaling.

In summary, our results demonstrate that related IQ motifs within the HECT E3 ubiquitin ligases UBE3B and UBE3C engage apo‐CaM through distinct molecular recognition mechanisms despite preserving the canonical features of the IQ motif sequence. Our biochemical, NMR, phylogenetic, and simulation analyses reveal that relatively subtle sequence differences reshape the stability, geometry, and dynamics of apo‐CaM recognition. These findings support a model where IQ motifs function as adaptable CaM‐binding elements whose interaction properties can diverge substantially during evolution while preserving canonical sequence features. More broadly, this work highlights how local sequence variation within conserved regulatory motifs can generate distinct signaling behaviors among closely related proteins.

## 4 MATERIALS AND METHODS

### 4.1 Peptide preparation

UBE3B and UBE3C IQ motif peptides corresponding to residues 29–58 of human UBE3B (UniProt Q7Z3V4) and residues 45–74 of human UBE3C (UniProt Q15386) were synthesized at >95% purity by GenScript. Canonical isoform 1 sequences were used for UBE3B and UBE3C. Lyophilized peptides were dissolved in experimental buffer prior to use.

### 4.2 Expression and purification of CaM constructs

Full‐length CaM, the isolated CaM *N*‐lobe (residues 1–81), and the isolated CaM *C*‐lobe (residues 76–148) were overexpressed in *Escherichia coli* BL21 (DE3) and purified using phenyl Sepharose or anion exchange chromatography followed by size‐exclusion chromatography on a Superdex 75 column using ÄKTA Fast Protein Liquid Chromatography systems, as previously described (Spratt et al., 2006). Size‐exclusion chromatography was performed in gel filtration buffer containing 50 mM Tris, 150 mM NaCl, and 1 mM ethylenediaminetetraacetic acid (EDTA) at pH 7.5. Purified CaM constructs were verified by electrospray ionization mass spectrometry. Uniformly ^13^C/^15^N‐labeled CaM was prepared in M9 minimal medium containing U‐^13^C‐glucose and ^15^NH_4_Cl as the sole carbon and nitrogen sources for NMR experiments. Purified CaM constructs were exchanged into Ca^2+^‐free NMR buffer containing 20 mM MES, 100 mM KCl, and 1 mM EDTA at pH 6.0 before NMR experiments.

### 4.3 ITC

ITC experiments were performed using an Affinity Isothermal Titration Calorimeter Low Volume with Automated Sample Changer (TA Instruments). Apo‐CaM constructs and synthetic UBE3B or UBE3C IQ peptides were prepared in matched Ca^2+^‐free ITC buffer containing 25 mM HEPES, 100 mM KCl, and 1 mM EDTA at pH 7.5. Synthetic IQ peptides ranging from 200 to 275 μM were titrated into 15–30 μM apo‐CaM constructs at 25°C. Raw injection heats were integrated and fit using a one‐site binding model. Buffer‐into‐buffer control titrations were used for background correction during data processing. Peptide‐into‐buffer control titrations were not performed. Each interaction was measured in triplicate.

### 4.4 NMR spectroscopy and CSP analysis

^1^H‐^15^N HSQC spectra were collected at 25°C for 300 μM ^15^N‐labeled apo‐CaM alone and following titration with UBE3B or UBE3C IQ peptides in NMR buffer containing 20 mM MES, 100 mM KCl, and 1 mM EDTA at pH 6.0. For the UBE3C IQ peptide titration, spectra were collected at peptide:apo‐CaM molar ratios of 0:1, 0.125:1, 0.25:1, 0.375:1, 0.5:1, 0.625:1, 0.75:1, 0.875:1, 1:1, 1.25:1, 2:1, and 4:1. For the UBE3B IQ peptide titration, spectra were collected at peptide:apo‐CaM molar ratios of 0:1, 0.125:1, 0.25:1, 0.375:1, 0.43:1, 0.5:1, 0.625:1, 0.75:1, 1:1, 1.25:1, 1.5:1, and 2:1.

NMR experiments were performed on a 600 MHz Varian INOVA spectrometer equipped with four channels, a deuterium decoupling accessory, and an enhanced HCN cold probe with XYZ axial gradients, housed and maintained in the Gustaf H. Carlson School of Chemistry and Biochemistry at Clark University. Backbone assignments for apo‐CaM/IQ complexes were generated in‐house using uniformly ^13^C/^15^N‐labeled apo‐CaM and standard triple‐resonance NMR experiments: 3D HNCACB (Grzesiek & Bax, 1992a), 3D CBCA(CO)NH (Grzesiek & Bax, 1992b), 3D HNCA (Grzesiek & Bax, 1992c; Kay et al., 2011; Muhandiram & Kay, 1994), 3D HN(CO)CA (Bax & Ikura, 1991; Grzesiek & Bax, 1992c), 3D HN(CA)CO (Clubb et al., 1992), and 3D HNCO (Grzesiek & Bax, 1992c; Kay et al., 2011; Muhandiram & Kay, 1994). Spectra were processed using NMRFx (Koag et al., 2025) and analyzed using NMRViewJ (Johnson, 2004; Johnson & Blevins, 1994).

CSPs were calculated for assigned residues using:

$$\Sigma \Delta \delta =|{\Delta \delta }^{1}H|+0.2|{\Delta \delta }^{15}N|$$

as previously described (Spratt et al., 2012). This formula was applied consistently to both datasets to compare relative perturbation patterns and map affected residues across the CaM sequence. Residues exhibiting significant perturbations were mapped onto AF3 (Abramson et al., 2024) or MD‐derived structural models of the corresponding apo‐CaM/IQ complexes.

Quantitative NMR binding analysis of the UBE3C titration was performed using a 1:1 finite‐concentration binding model with the apo‐CaM concentration fixed at 300 μM (Hobbs et al., 2022; Williamson, 2013). Concentration‐dependent chemical‐shift trajectories for G113, L116, D131, A147, and K148 were fit globally with a shared *K*_d_ and residue‐specific free‐ and bound‐state chemical shifts (Hobbs et al., 2022; Williamson, 2013). Reported uncertainty represents the standard deviation of *K*_d_ estimates obtained by residual bootstrap resampling of the global fit (Efron & Tibshirani, 1986). For the UBE3B titration, the 300 μM apo‐CaM concentration was substantially higher than the submicromolar affinity measured independently by ITC. Under these conditions, the NMR titration was not sufficiently sensitive to derive an independent quantitative *K*_d_.

### 4.5 AF3 structural modeling

Structures of the UBE3B IQ/apo‐CaM and UBE3C IQ/apo‐CaM complexes were predicted using AF3 (Abramson et al., 2024) through the AlphaFold Server (https://alphafoldserver.com). Five predicted structures were generated for each complex. The CaM sequence was taken from the Protein Data Bank (PDB ID: 1CFD; Kuboniwa et al., 1995). The 30‐residue IQ motifs were derived from residues 29–58 of UBE3B (UniProt ID: Q7Z3V4) and residues 45–74 of UBE3C (UniProt ID: Q15386), respectively. Canonical isoform 1 sequences were used for UBE3B and UBE3C. Predicted models were evaluated using predicted local distance difference test confidence scores, and models exhibiting high overall confidence were selected for MD simulations.

### 4.6 MD simulations

The *N*‐ and *C*‐termini of the IQ motif were capped with the ACE and NME groups, respectively, while the termini of apo‐CaM were charged. The ff14SB force field (Maier et al., 2015) was employed. To neutralize the system, 20 K^+^ counter ions were added. The protein complex was then solvated using the TIP3P (Jorgensen et al., 1983) explicit water model, with all protein atoms placed at least 15 Å from the edges of the water box. The KCl concentration was set to 0.1 M to match the experimental conditions.

MD simulations were carried out using the AMBER18 package (Case et al., 2018). Two rounds of energy minimization were carried out: 2000 steps with positional restraints (force constant = 50.0 kcal/(mol·Å^2^)) on all protein atoms, followed by another round of 2000‐step minimization without restraints. In each round, 100 steps of the steepest descent method and the subsequent 1900 steps of the conjugate gradient method were employed. The equilibration was conducted in four steps: 1 ns equilibration in the NVT ensemble at 10 K and 1 bar with the same restraints as applied during minimization; 1 ns equilibration in the NPT ensemble under the same conditions; 1 ns equilibration in the NPT ensemble at 300 K and 1 bar with the same restraints; and the final 10 ns equilibration in the NPT ensemble under the same conditions without any restraints. Temperature and pressure were regulated using the Berendsen method (Berendsen et al., 1984) with a coupling constant of 1 ps. The production run was carried out in the NPT ensemble at 300 K and 1 bar. Constraints were applied to all the bonds involving hydrogen atoms using the SHAKE algorithm (Ryckaert et al., 1977). The cutoff for the short‐range nonbonded interactions was set to 8 Å, while the particle mesh Ewald method (Crowley et al., 1997; Darden et al., 1993; Essmann et al., 1995; Sagui & Darden, 1999) was used to treat the interactions beyond this cutoff. The time step was set to 2 fs. Periodic boundary conditions were applied. Three independent simulations were carried out for each complex with a snapshot saved every 20 ps for a total of 2 μs per trajectory.

### 4.7 MD trajectory analysis

The RMSD and root‐mean‐square fluctuation were computed using visual molecular dynamics (VMD) (Humphrey et al., 1996). The radius of gyration (Rg) for all heavy atoms was calculated using the CPPTRAJ program (Roe & Cheatham 3rd, 2013). A CaM residue and a residue of the IQ motif are considered in contact if any pair of their heavy atoms is within 4.5 Å. If a given pair of residue contacts was present in at least 75% of the total snapshots, then this pair of residues was considered in contact in the simulations. The solvent‐accessible surface area (SASA) was computed using VMD with a 1.4 Å probe radius. The BSA between CaM and the IQ motif was defined as:

$$\mathrm{BSA}={\text{SASA}}_{\mathrm{CaM}}+{\text{SASA}}_{\mathrm{IQ}}-\;{\text{SASA}}_{\text{complex}}$$

The Timeline plugin of VMD was used to analyze the helicity of the IQ motif. Interhelical distance was measured as the minimal distance between every pair of Cα atoms in the two helices under consideration. The R package (version 4.1.0) was used for the Kolmogorov–Smirnov test and Hartigan's dip test (Team RC, 2021). For a bimodal distribution, the local minimum was identified as the separation point, and then the data were split into two subgroups to calculate the median for each of them. For all analyses in this study, the three independent trajectories of each complex were concatenated after removing the first 250 ns from each trajectory.

### 4.8 Phylogenetic analysis

IQ motif sequences from 450 representative IQ‐containing proteins were downloaded from UniProt, and the IQ domain was extracted using HMMER v3.4 (Potter et al., 2018). The IQ domain was aligned using MAFFT v7.0 (i‐INS‐I algorithm) (Katoh et al., 2002; Katoh & Standley, 2013). Phylogenetic relationships were reconstructed to compare the evolutionary relationships of the UBE3B and UBE3C IQ motifs with previously characterized apo‐CaM/IQ interactions using IQ‐TREE 2 (Minh et al., 2020) with 10,000 rapid bootstrap replicates and an automatic model‐finding algorithm. Phylogenetic trees were visualized and annotated using iTOL (Letunic & Bork, 2024).

## AUTHOR CONTRIBUTIONS

**Emily G. Schaffter:** Conceptualization; investigation; writing – original draft; methodology; validation; visualization; writing – review and editing; formal analysis. **Xiaohuan Lu:** Conceptualization; investigation; writing – original draft; methodology; validation; visualization; writing – review and editing; software; formal analysis. **Anna E. Tinklenberg:** Investigation; writing – original draft; methodology; validation; visualization; writing – review and editing; formal analysis. **Megan G. Hill:** Conceptualization; investigation; methodology; formal analysis. **Rafael H. Levin:** Conceptualization; investigation; methodology; formal analysis. **Javier F. Tabima:** Investigation; software; writing – original draft; writing – review and editing; formal analysis; methodology. **Shuanghong Huo:** Conceptualization; investigation; writing – original draft; writing – review and editing; visualization; validation; methodology; software; formal analysis; supervision. **Donald E. Spratt:** Project administration; supervision; conceptualization; investigation; funding acquisition; writing – original draft; writing – review and editing; visualization; validation; methodology; formal analysis.

## Supporting information


**Supplementary Figure S1:** Ubiquitin protein ligase E3B (UBE3B) and ubiquitin protein ligase E3C (UBE3C) isoleucine‐glutamine (IQ) motifs occupy distinct branches within an IQ motif phylogeny. A subset of an IQ motif phylogenetic tree constructed from approximately 450 IQ motif sequences identified using the PROSITE IQ motif profile (PS50096) within the InterPro database (Paysan‐Lafosse et al., 2023; Sigrist et al., 2013). Red boxes indicate the positions of the UBE3C IQ motif (top) and UBE3B IQ motif (bottom), highlighting their placement in distinct regions of the phylogeny.

**Supplementary Table S1:** Structural diversity of apo‐calmodulin/IQ motif recognition modes.

## Data Availability

The data that support the findings of this study are available from the corresponding author upon reasonable request.
