# Supplementary material for: Conserved IQ motifs, divergent apo‐calmodulin recognition in UBE3B and UBE3C
Source: Protein Sci. 2026 Oct 23;35(11):e70826. doi: 10.1002/pro.70826 (PMC13693507; doi:10.1002/pro.70826)

Supplemental Figure S1

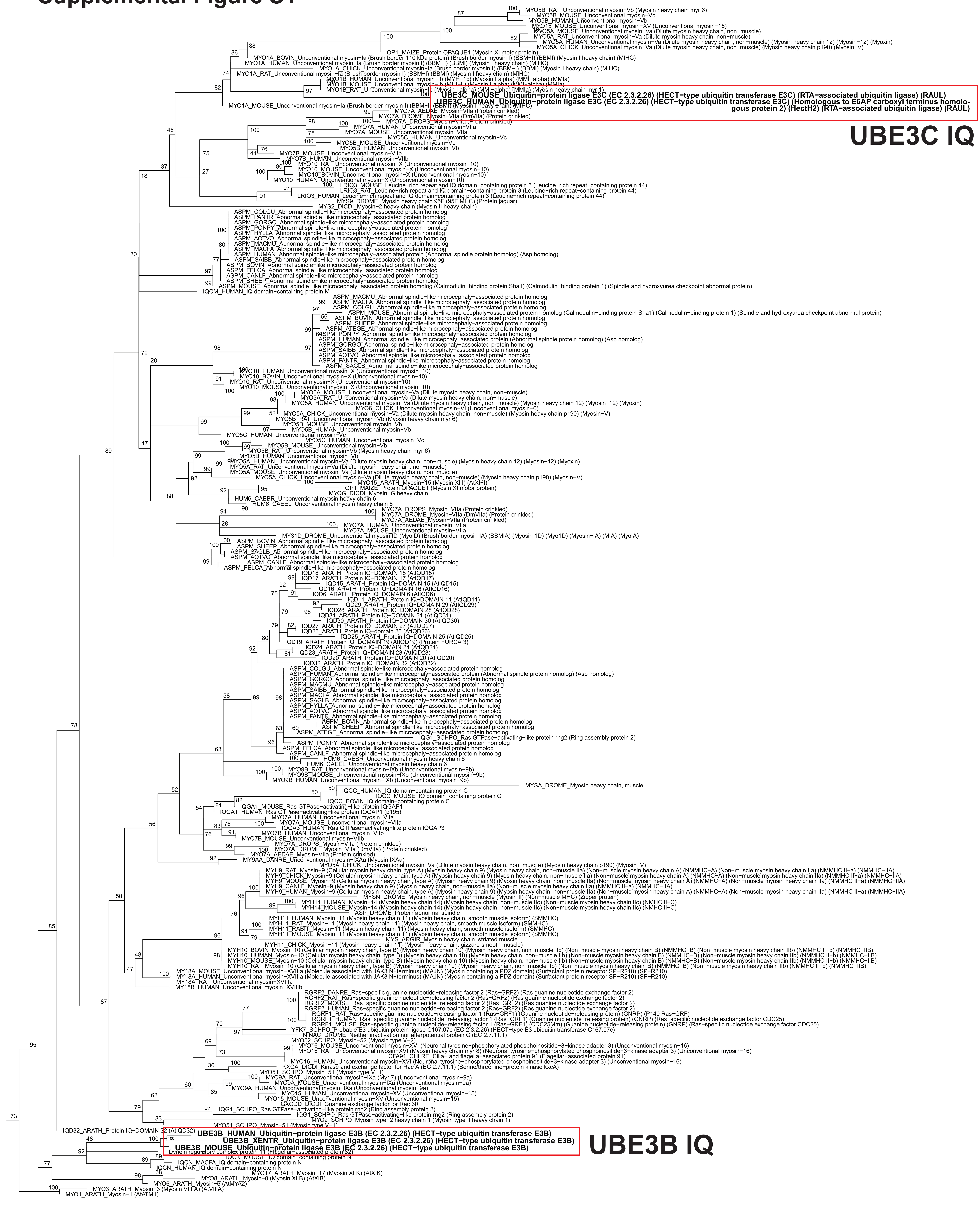

Supplement: Supplementary file 1 [file PRO-35-e70826-s001.pdf]
