# Supplementary material for: Conserved IQ motifs, divergent apo‐calmodulin recognition in UBE3B and UBE3C
Source: Protein Sci. 2026 Oct 23;35(11):e70826. doi: 10.1002/pro.70826 (PMC13693507; doi:10.1002/pro.70826)
Supplement: Supplementary file 2 [file PRO-35-e70826-s002.docx]

**Supplementary Table S1 – Structural diversity of apo-calmodulin/IQ motif recognition modes**

| IQ-Containing Protein | Protein Abbreviation | IQ Motif Residues | Reported CaM Binding Affinity (nM) | Binding Affinity Method | PDB ID *BMRB ID*  & Reference | Structural Method | Resolution (Å) | Apo-CaM/IQ Conformation |
| --- | --- | --- | --- | --- | --- | --- | --- | --- |
| IQ motif and SEC7 domain-containing protein 1 | IQSEC1 | 141-154 |  |  | 7VMB (70) | X-Ray | 2.00 | Extended |
| Neurogranin | Ng | 32-46 | 43.5 | ITC | 4E50 (71) | X-Ray | 2.70 | Extended |
| Neuromodulin | Nm | 38-51 | 127 | ITC | 4E53 (71) | X-Ray | 2.69 | Extended |
| Sodium channel protein type 2 subunit alpha | Na_V_1.2 | 1912-1925 | 6.3 | FRET | 6BUT *27095* (72) | NMR |  | Extended |
| Sodium channel protein type 4 subunit alpha | Na_V_1.4 | 1734-1747 | 17 ± 3 (1599-1764) 114 ± 11 (1599-1754) | ITC | 6MBA (73) | X-Ray | 1.80 | Extended |
| Sodium channel protein type 5 subunit alpha | Na_V_1.5 | 1908-1921 |  |  | 4DCK (74) | X-Ray | 2.20 | Extended |
|  |  |  | 46 ± 33 to 105 ± 15 (Buffer Dependent) | ITC | 4OVN (75) | X-Ray | 2.80 | Extended |
| Sodium channel protein type 8 subunit alpha | Na_V_1.6 | 1900-1913 | 2630 | ITC | 3WFN (76) | X-Ray | 1.95 | Extended |
| IQ Containing Protein G (Dynein regulatory complex protein 9) | IQCG | 400-413 | 5.07 | SPR | 4LZX (77) | X-Ray | 1.50 | Compact |
| Unconventional myosin-Ib | Myo1b | 711-724 |  |  | 6C1D (68) | Cryo-EM | 3.20 | Compact |
|  |  |  |  |  | 6C1H (68) | Cryo-EM | 3.90 | Compact |
|  |  |  |  |  | 6C1G (68) | Cryo-EM | 3.80 | Compact |
|  |  |  |  |  | 4L79 (78) | X-Ray | 2.30 | Compact |
|  |  |  |  |  | 5V7X (68) | X-Ray | 3.14 | Compact |
| Unconventional myosin-Ic | Myo1c | 741-754 |  |  | 4BYF (79) | X-Ray | 2.74 | Compact |
| Unconventional myosin-V | Myo5a | 773-786 & 796-809 |  |  | 2IX7 (27) | X-Ray | 2.50 | Compact |
| Unconventional myosin-VIIa | Myo7 | 843-856 |  |  | 5WSU (69) | X-Ray | 3.00 | Compact |
| Unconventional myosin-X | Myo10 | 749-762 & 772-785 |  |  | 5I0I (32) | X-Ray | 3.15 | Compact |
| Unconventional myosin-1c | Myo1c | 698-764 | IQ2 - 490; IQ3 - 3140 | ITC | 4R8G (31) | X-Ray | 3.50 | IQ1 and IQ2 - Compact;  IQ3 - Single Lobe |
| Purkinje cell protein 4 | PEP-19 | 46-59 |  |  | 2N77 *25796* (80) | NMR |  | Single Lobe |
| Sodium channel protein type 2 subunit alpha | Na_V_1.2 | 1912-1925 | ≤ 10 | Fluorescence Anisotropy | 2KXW (29) | NMR |  | Single Lobe |
| Sodium channel protein type 5 subunit alpha (isoform b variant) | Na_V_1.5 | 1908-1921 |  |  | 2L53 *17264* (28) | NMR |  | Single Lobe |
| Voltage-dependent L-type calcium channel subunit alpha-1C | Ca_V_1.2 | 1654-1667 |  |  | 6CTB *27530* | NMR |  | Single Lobe |
| Unconventional myosin-Va | Myo5a | All 6 IQ |  |  | 2DFS (81) | Electron Crystallography | 24.00 | Conformation Differs Based on IQ |
|  |  |  |  |  | 8OF8 (82) | Cryo-EM | 7.50 | Conformation Differs Based on IQ |
